# A Rare Case When Acromegaly Meets Cushing Syndrome

**DOI:** 10.1210/jcemcr/luad145

**Published:** 2023-12-19

**Authors:** Jacob Gabbay, Samantha Steinmetz-Wood, Natalia Chamorro-Pareja, Kaitlyn Barrett

**Affiliations:** Department of Medicine, University of Vermont Medical Center, Burlington, VT 05401, USA; Division of Endocrinology, Diabetes and Metabolism, Department of Medicine, University of Vermont Medical Center, South Burlington, VT 05403, USA; Neuroendocrine Unit, Massachusetts General Hospital, Boston, MA 02114, USA; Harvard Medical School, Boston, MA 02115, USA; Division of Endocrinology, Diabetes and Metabolism, Department of Medicine, University of Vermont Medical Center, South Burlington, VT 05403, USA

**Keywords:** acromegaly, adrenal Cushing syndrome, IGF monitoring

## Abstract

Acromegaly is very uncommon, as is non-iatrogenic Cushing syndrome; we discuss a patient who was found to have both a pituitary adenoma causing acromegaly and a cortisol-producing adrenal adenoma causing Cushing syndrome within 1 year. She was a healthy, 44-year-old woman who presented with visual changes and was found to have bitemporal hemianopsia and a 3.3-cm pituitary mass along with central hypogonadism, central hypothyroidism, and suppressed adrenocorticotropin and discrepant cortisol. After transsphenoidal resection she had declining, but persistently elevated, insulin-like growth factor 1 (IGF-1), raising concern for persistent acromegaly. She also was experiencing several cushingoid symptoms and was found to have elevated salivary and urinary cortisol. An abdominal computed tomography scan showed a 3.1-cm adrenal adenoma, and she subsequently underwent adrenalectomy. Following adrenalectomy, her cortisol levels normalized, and her IGF-1, growth hormone, and oral glucose tolerance test showed substantial improvement consistent with previous reports linking hypercortisolism and elevated IGF-1 levels. Combinations of pituitary and adrenal disease are seen in a handful of genetic syndromes; however, her clinical presentation and genetics do not fit with known syndromes. This case describes two rare endocrine tumors in one patient and associated limitations of routine laboratory testing.

## Introduction

Acromegaly resulting from a pituitary adenoma is a very uncommon occurrence, with roughly 30 to 70 individuals affected per million people [1]. Cushing syndrome from an adrenal adenoma is even rarer, with approximately 0.6 individuals affected per million people [2]. Based on these statistics, the chances of both co-occurring in an individual patient would be astronomically low; however, several genetic syndromes can link these 2 diseases, including multiple endocrine neoplasia type 1 (MEN1), multiple endocrine neoplasia type 4, Carney complex, and McCune-Albright syndrome.

In addition to the challenge of diagnosing both conditions simultaneously, the presence of both causes a laboratory dilemma. Biochemical control following treatment of a somatotroph adenoma is characterized by growth hormone (GH) suppression during an oral glucose tolerance test (OGTT) and normalization of age-adjusted insulin-like growth factor 1 (IGF-1) levels [3]. There is limited information on how hypercortisolism affects these tests, but there are some study data that suggest patients with Cushing syndrome have elevated IGF-1 compared to controls [4, 5]. Patients with multiorgan endocrine involvement need to be monitored for confounders in laboratory testing.

## Case Presentation

**Figure 1.**
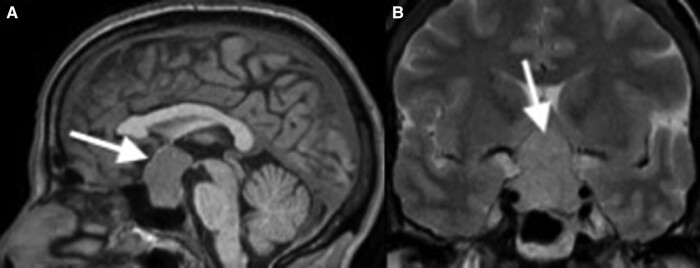
Initial magnetic resonance imaging scan of the pituitary showing a large 2.1 × 2.5 × 3.3-cm macroadenoma with invasion into the right cavernous sinus and with upward displacement and flattening of the optic chiasm. A, Sagittal view. B, Coronal view.

A 44-year-old woman presented to her optometrist for progressive blurring of vision and was found to have bitemporal hemianopsia. Her medical history consisted of only mild hypertension for which she was not taking any medications. She reported no contributory family history. A pituitary magnetic resonance imaging scan was obtained showing a 2.1 × 2.5 × 3.3-cm macroadenoma with invasion into the right cavernous sinus, and with upward displacement and flattening of the optic chiasm (Fig. 1).

She had significant elevation of IGF-1 to 610 ng/mL (79.7 nmol/L) (49-240 ng/mL; 12.3-31.4 nmol/L), with an elevated GH level of 23 ng/mL (23 μg/L) (0.01-3.61 ng/mL; 0.01-3.61 μg/L) consistent with acromegaly. She also had coexisting pituitary deficiencies including hypogonadotropic hypogonadism and central hypothyroidism (Table 1, column 2). Morning adrenocorticotropin (ACTH) was low at less than 5.0 pg/mL (<1.1 pmol/L) (7.2-63 pg/mL; 1.6-13.9 pmol/L) on multiple occasions with morning cortisol within normal limits at 18 μg/dL (497 nmol/L) (4-23 μg/dL; 111-1630 nmol/L). When the patient was seen by endocrinology she confirmed several symptoms and signs consistent with acromegaly including amenorrhea, arthralgias, skin tags, coarsening facial features, increased teeth spacing, hair thinning, increased ring and shoe size, hair loss, and new prediabetes. She also reported a 30-pound weight gain, but lost most of this weight by following an intensive weight-loss program consisting of a restrictive diet. Her menstrual periods ceased only 2 months prior to presentation. She associated most of her symptoms to changes in her routine and stressors during the COVID-19 pandemic and did not seek medical evaluation early for these symptoms. Due to uncertainty regarding her low ACTH level in conjunction with normal cortisol levels, she was started on perioperative glucocorticoids as well as thyroid replacement. She underwent a transsphenoidal resection of her pituitary macroadenoma, and the surgical pathology revealed a sparsely granulated somatotroph adenoma that was PIT1 positive, GH weakly positive, keratin cAM 5.2 positive, Ki67 index 1.2%, and was negative for prolactin, ACTH, and TPIT. She had repeat laboratory tests after a hydrocortisone taper was completed (see Table 1, column 3).

**Table 1.** Pituitary laboratory values before intervention, 6 weeks after pituitary resection, and 4 weeks after adrenalectomy (6 months after pituitary resection)

Parameter	Initial labs	6 weeks post pituitary resection	4 weeks post adrenalectomy*	Reference range (non-pregnant premenopausal females)
ACTH	**< 5.0 pg/mL** **(1.1 pmol/L)**	**< 5.0 pg/mL** **(1.1 pmol/L)**	**< 5.0 pg/mL** **(1.1 pmol/L)**	7.2-63 pg/mL (1.6-13.9 pmol/L)
Serum AM Cortisol	18 μg/dL (497 nmol/L)	16 μg/dL (443 nmol/L)	**<1 μg/dL** **(27.6 nmol/L)**	4-23 μg/dL (111-1630 nmol/L)
LH	**<0.3 mIU/mL** **(0.3 IU/L)**	**<0.3 mIU/mL** **(0.3 IU/L)**	6.3 mIU/mL (6.3 IU/L)	0.5-76.3 mIU/mL (0.5-76.3 IU/L)
FSH	**<0.3 mIU/mL** **(0.3 IU/L)**	**<0.3 mIU/mL** **(0.3 IU/L)**	1.8 mIU/mL (1.8 IU/L)	1.5-33.4 mIU/mL (1.5-33.4 IU/L)
Estradiol	**<12 pg/mL** **(44 pmol/L)**	**<12 pg/mL** **(44 pmol/L)**	40 pg/mL (146.8 pmol/L)	17-200 pg/mL (62.4-734 pmol/L)
GH	**23 ng/mL** **(23 μg/L)**	1.81 ng/mL (1.81 μg/L)	0.88 ng/mL (0.88 μg/L)	0.01-3.61 ng/mL (0.01-3.61 μg/L)
OGTT GH		**0.49 ng/mL** **(0.49 μg/L)**	0.2 ng/mL (0.2 μg/L)	0.01-0.4 ng/mL ** (0.01-0.4 μg/L)
IGF 1	**610 ng/mL** **(79.7 nmol/L)**	**385 ng/mL** **(50.3 nmol/L)**	**290 ng/mL** **(37.9 nmol/L)**	49-240 ng/mL (12.3-31.4 nmol/L)
Prolactin	9.1 ng/mL (9.1 μg/L)	3.9 ng/mL (3.9 μg/L)		2.8-29.2 ng/mL (2.8-29.2 μg/L)
TSH	1.69 mIU/L (1.69 μIU/mL)	**0.29 mIU/L** **(0.29 μIU/mL)**	**0.05 mIU/L** **(0.05 μIU/mL)**	0.47-4.68 mIU/L (0.47-4.68 μIU/mL)
T4, free	**0.7 ng/dL** **(9.0 pmol/L)**	0.8 ng/dL (10.3 pmol/L)	1.5 ng/dL (19.4 pmol/L)	0.8-2.2 ng/dL (10.3-28.4 pmol/L)

Abnormal values are shown in bold font. Values in parenthesis are International System of Units (SI).

Abbreviations: ACTH, Adrenocorticotropic hormone; LH, luteinizing hormone; FSH, follicular stimulating hormone; GH, growth hormone; IGF, insulin like growth factor; TSH, thyroid stimulating hormone; T4, thyroxine.

*6 months post pituitary resection.

**2 hours following glucose load.

Her IGF-1 had improved to 385 ng/mL (50.3 nmol/L) (49-240 ng/mL; 12.3-31.4 nmol/L) but it was still elevated, her gonadotropins were still undetectable, and her ACTH was still suppressed with normal morning cortisol levels. She also had a robust response on an ACTH stimulation test. A 3-month postoperative pituitary magnetic resonance imaging scan showed a lobular, hypoenhancing soft tissue in the dorsal aspect of the sella concerning for possible residual tumor. By this time, her most recent IGF-1 was still elevated at 427 ng/mL (55.8 nmol/L). Her 2-hour OGTT using ultrasensitive GH suppressed partially from 1.01 ng/mL (1.01 μg/L) to 0.49 ng/mL (0.49 μg/L) (0.01-0.4 ng/mL; 0.01-0.4 μg/L 2 hours following glucose load) (Table 2). She continued to complain of symptoms such as weight gain, poor sleep, and facial and ankle swelling. Testing for hypercortisolism was finally undertaken and was consistent with Cushing syndrome with midnight salivary cortisol elevated more than 4 times the upper limit of normal on 3 successive tests: 730 ng/dL, 502 ng/dL, 404 ng/dL (2012 nmol/L, 1384 nmol/L, 1114 nmol/L] (<100 ng/dL; < 276 nmol/L). She was also found to have elevated 24-hour urinary free cortisol of 817 μg/24 hours (2254 nmol/day) (3.5-45 μg/24 hours; 9.7-124.2 nmol/day) (Table 3). Dehydroepiandrosterone sulfate was low at less than 5 μg/dL (0.13 μmol/L) (75-410 μg/dL; 1.95-10.66 μmol/L).

**Table 2.** Oral glucose tolerance tests performed 3 months post transsphenoidal surgery and 6 weeks post adrenalectomy (6 months post transsphenoidal surgery)

	Post pituitary resection (3 mo)		Post adrenalectomy (6 weeks)*	
	Glucose	GH	Glucose	GH
Initial	107 mg/dL (5.93 mmol/L)	1.01 ng/mL (1.01 μg/L)	79 mg/dL (4.38 mmol/L)	0.88 ng/mL (0.88 μg/L)
30 min			129 mg/dL (7.16 mmol/L)	0.34 ng/mL (0.34 μg/L)
60 min			164 mg/dL (9.16 mmol/L)	0.29 ng/mL (0.29 μg/L)
90 min			165 mg/dL (9.16 mmol/L)	0.21 ng/mL (0.2 μg/L)
120 min	149 mg/dL (8.27 mmol/L)	**0.49 ng/mL** **(0.49 μg/L)**	142 mg/dL (7.88 mmol/L)	0.20 ng/mL (0.20 μg/L)

Reference GH 2 hours after glucose load 0.01 -0.4 ng/mL (0.01 -0.4 μg/L).

Abnormal values are shown in bold font. Values in parenthesis are International System of Units (SI).

Abbreviations: GH, Growth Hormone.

*6 months post pituitary resection.

**Table 3.** Initial testing for hypercortisolism

Parameter	Value	Reference range
Midnight salivary cortisol	**730 ng/dL (2012 nmol/L)** **502 ng/dL (1384 nmol/L)** **404 ng/dL (1114 nmol/L)**	<100 ng/dL (276 nmol/L)
24-h urine free cortisol	**817 mcg/24 h** **(2254 nmol/day)**	3.5-45 mcg/24 h (9.7-124.2 nmol/day)
DHEA sulfate	**<5 μg/dL** **(0.13 μmol/L)**	75-410 μg/dL (1.95-10.66 μmol/L)

Abnormal values are shown in bold font.

Values in parenthesis are International System of Units (SI).

Abbreviations: DHEA, Dehydroepiandrosterone.

**Figure 2.**
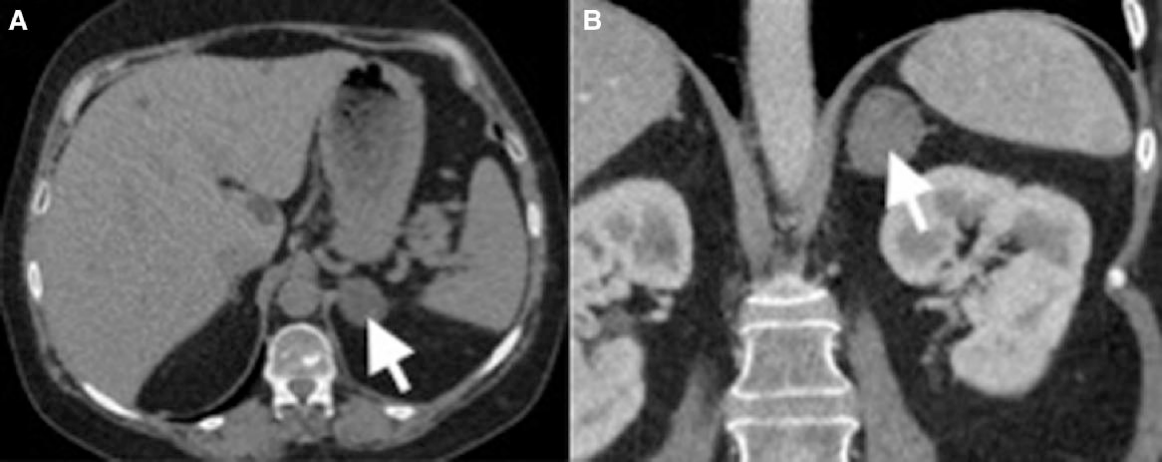
Computed tomography of the abdomen showing a 3.1-cm, lipid-rich adrenal adenoma (5 Hounsfield units). A, Axial view. B, Coronal view.

A computerized tomography scan of the abdomen was obtained showing a 3.1-cm, lipid-rich left adrenal adenoma (5 Hounsfield units), which confirmed the likely source of her coexisting adrenal Cushing syndrome (Fig. 2).

## Treatment

The patient underwent laparoscopic left adrenalectomy with pathology consistent with adrenal adenoma.

## Outcome and Follow-up

In the following weeks after surgery, the patient reported considerable symptomatic improvement including better sleep, improved ankle swelling, and weight loss. To our astonishment, follow-up laboratory tests in the following months showed recovery of the hypothalamic-pituitary-gonadal axis (see Table 1, column 4), with return of menses, and improvement in the IGF-1 level to normal levels, and successful suppression of GH level on a 2-hour OGTT: 0.88 ng/mL to 0.20 ng/mL (0.88 μg/L to 0.20 μg/L). This was consistent with biochemical remission of acromegaly (see Table 2).

Despite hydrocortisone wean attempts, the patient's hypothalamic-pituitary-adrenal axis has not recovered fully to date. However, there is promising early detection of the ACTH level, which is no longer suppressed. The patient did undergo genetic testing and is heterozygous for a variant of unknown significance detected in the *PTCH1* gene, which has no established connection to the development of acromegaly or adrenal Cushing syndrome.

## Discussion

The definition of biochemical control of acromegaly after resection has been redefined over time [6]. Broadly, it is defined as GH suppression during an OGTT and normalization of age-adjusted IGF-1 level 3 to 6 months postoperatively [6, 7]. However, it has been estimated that up to 30% of patients could have discrepant results between IGF-1 and GH levels. Suppression was traditionally defined as GH nadir of less than 1.0 ng/mL (<1.0 μg/L) after OGTT, but now with ultrasensitive assays, the consensus for suppression is a GH level of less than 0.4 ng/mL (<0.04 μg/L) [6]. There was also a retrospective study of postsurgical patients with a mean follow-up of 39 months that suggests a 3-month IGF-1 level less than 1.25 times the upper limit of normal is associated with long-term remission [3]. There are also other factors that can affect GH and IGF-1 levels, such as pregnancy, diabetes, oral estrogen (not transdermal estrogen), and critical illness [7]. There is limited literature on the effects of hypercortisolism on IGF-1 levels, but higher IGF-1 levels were identified in patients with Cushing syndrome as early as 1993 [4]. There was also a retrospective case-control study, published in 2019, measuring preoperative and postoperative IGF-1 levels in Cushing disease patients to matched controls that found a significantly higher proportion of Cushing patients with elevated serum IGF-1 above the reference range compared to controls [5]. In addition, among the patients who achieved remission of their Cushing, IGF-1 levels decreased significantly postoperatively. Though this study did not involve acromegaly patients and included patients with pituitary Cushing disease rather than adrenal Cushing syndrome, we observed in our case a similar phenomenon by which the IGF-1 normalized after cure of the overt hypercortisolism.

This is a rare case, but similar cases have been reported, the most similar of which was published in 2011 and detailed the presentation of a patient with acromegaly who was found to have hypercortisolism from an adrenal adenoma 6 years after the resection of her pituitary adenoma. The authors reported that, post adrenalectomy, the patient needed significantly less pegvisomant for biochemical control of her acromegaly [8].

Our case is different in that our patient had overt Cushing syndrome, whereas this patient had mild autonomous cortisol secretion. Because of suppressed ACTH and the focus on GH excess in association with a macroadenoma, we did not initially look for cortisol excess as a cause of our patient’s symptoms. Also, because most visits were conducted by televideo during this time, and there were still COVID restrictions and more limited access in the health care setting, not all clinical features were immediately evident, and in addition, the overlap of symptoms for these 2 different conditions made the diagnosis challenging.

During our patient’s workup, genetic testing was completed to identify a unifying genetic syndrome explaining the co-occurrence of 2 different endocrine tumors. Here genes were analyzed using next-generation sequencing and Sanger sequencing. During analysis, the coding domains and a portion of flanking regions were searched; notably, the promotor genes and a portion of the untranslated regions were not reported. The only remarkable finding in her genes was *PTCH1*, a variant of unknown significance that has no reported relationships to acromegaly or Cushing syndrome. The known syndrome that overlaps the most with her disease would be MEN1 syndrome. Most defects in the *MEN1* gene would have been caught with this approach to analysis; however, a portion of the promoter and untranslated genes was not reported. Genes in these regions may be responsible for 5% to 25% of *MEN1* gene dysfunction [9]. However, she had no evidence of primary hyperparathyroidism, which has a high penetrance in MEN1 and is most often the first presentation in MEN1 (>93% penetrance and first manifestation in >67%) [10]. Carney complex is another disease process that could be considered, but our patient did not have primary pigmented nodular adrenocortical disease. McCune-Albright syndrome is unlikely given that she had no fibrous dysplasia of bone or café au lait skin macules on examination.

This case raises the question of whether we should be actively searching for additional endocrine abnormalities in patients diagnosed with one endocrine problem. We believe that actively searching for other endocrine abnormalities should be evaluated on a case-by-case basis. While identifying other abnormalities early could be lifesaving in a subset of cases, it comes at a cost. Endocrine testing in patients with dysfunction of another endocrine organ may be difficult to interpret, and false positives could result in unnecessary invasive testing. Given the questionable net benefit, each patient should be evaluated on a case-by-case basis. As genetic testing continues to improve, we suspect there may be additional subsets of patients who warrant further evaluation. Overall, this case serves as a reminder that we should keep a high index of suspicion for concomitant endocrine abnormalities and in those cases our gold-standard testing may be insufficient.

## Learning Points

- In patients with rare endocrine tumors, consider a genetic endocrine syndrome, as other endocrine tumors are more common in these patients than in the general population.
- Gold-standard testing can be undermined by metabolic or physiologic abnormalities.
- While televideo visits are a tremendous asset to medicine, at times the lack of a comprehensive physical exam can limit appropriate evaluation.

## Contributors

All authors made individual contributions to authorship. S.S.W., N.C., and K.B. were involved in the diagnosis and management of this case and text editing. J.G. and S.S.W. were involved in manuscript preparation and submission. J.G. was involved in table and figure preparation. All authors reviewed and approved the final draft.

## Data Availability

Original data generated and analyzed for this case report are included in this published article. The laboratory that performed the genetic testing described (Ambry Genetics) deposits variant-level data to ClinVar (http://www.clinvar.com/), a public repository that aggregates information about human genomic variation.

## Abbreviations

ACTH: adrenocorticotropin
GH: growth hormone
IGF-1: insulin-like growth factor 1
MEN1: multiple endocrine neoplasia type 1
OGTT: oral glucose tolerance test
